# Supplementary material for: Relationship between cognitive abilities and mental health as represented by cognitive abilities at the neural and genetic levels of analysis
Source: eLife. 2025 Nov 14;14:RP105537. doi: 10.7554/eLife.105537 (PMC12618009; doi:10.7554/eLife.105537)
Supplement: Supplementary file 1. — Med = Median IQR = interquartile range; CV = Coefficient of variation; CBCL = Child Behavioural Checklist, reflecting children’s emotional and behavioural problems; UPPS-P = Urgency, Premeditation, Perseverance, Sensation seeking, and Positive urgency Impulsive Behaviour Scale; BAS = Behavioural Activation System. Under the variable names, there are information about the method to compute these variables and the original variables names in ABCD data dictionary. [file elife-105537-supp1.docx]

Supplementary ﬁle 1. Summary statistics of the measures of ental health in the baseline. Med = Median IQR = interquartile range; CV = Coefficient of variation; CBCL = Child Behavioural Checklist, reflecting children’s emotional and behavioural problems; UPPS-P = Urgency, Premeditation, Perseverance, Sensation seeking and Positive urgency Impulsive Behaviour Scale; BAS = Behavioural Activation System. Under the variable names, there are information about the method to compute these variables and the original variables names in ABCD data dictionary.

| No | Variable | Stats / Values | Graph | Valid |
| --- | --- | --- | --- | --- |
| 1 | Anxious/Depressed CBCL [numeric]  CBCL_SCR_SYN_ANXDEP_R | Mean (sd) : 2.5 (3.1) min < med < max: 0 < 1 < 26 IQR (CV) : 4 (1.2) | 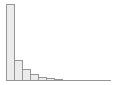 | 11754 (99.9%) |
| 2 | Withdrawn CBCL [numeric]  CBCL_SCR_SYN_WITHDEP_R | Mean (sd) : 1 (1.7) min < med < max: 0 < 0 < 15 IQR (CV) : 1 (1.7) | 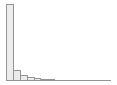 | 11754 (99.9%) |
| 3 | Somatic CBCL [numeric]  CBCL_SCR_SYN_SOMATIC_R | Mean (sd) : 1.5 (2) min < med < max: 0 < 1 < 16 IQR (CV) : 2 (1.3) | 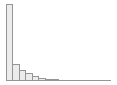 | 11754 (99.9%) |
| 4 | Social CBCL [numeric]  CBCL_SCR_SYN_SOCIAL_R | Mean (sd) : 1.6 (2.3) min < med < max: 0 < 1 < 18 IQR (CV) : 2 (1.4) | 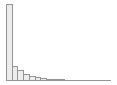 | 11754 (99.9%) |
| 5 | Thought CBCL [numeric]  CBCL_SCR_SYN_THOUGHT_R | Mean (sd) : 1.6 (2.2) min < med < max: 0 < 1 < 18 IQR (CV) : 2 (1.4) | 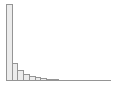 | 11754 (99.9%) |
| 6 | Attention CBCL [numeric]  CBCL_SCR_SYN_ATTENTION_R | Mean (sd) : 3 (3.5) min < med < max: 0 < 2 < 20 IQR (CV) : 5 (1.2) | 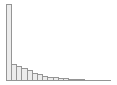 | 11754 (99.9%) |
| 7 | Rule-Breaking CBCL [numeric]  CBCL_SCR_SYN_RULEBREAK_R | Mean (sd) : 1.2 (1.9) min < med < max: 0 < 0 < 20 IQR (CV) : 2 (1.6) | 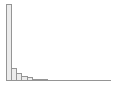 | 11754 (99.9%) |
| 8 | Aggressive CBCL [numeric]  CBCL_SCR_SYN_AGGRESSIVE_R | Mean (sd) : 3.3 (4.4) min < med < max: 0 < 2 < 36 IQR (CV) : 5 (1.3) | 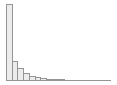 | 11754 (99.9%) |
| 9 | Negative Urgency UPSS [numeric]  UPPS_Y_SS_NEGATIVE_URGENCY | Mean (sd) : 8.5 (2.6) min < med < max: 4 < 8 < 16 IQR (CV) : 3 (0.3) | 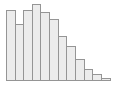 | 11739 (99.8%) |
| 10 | Lack of Planning UPSS [numeric]  UPPS_Y_SS_LACK_OF_PLANNING | Mean (sd) : 7.7 (2.4) min < med < max: 4 < 8 < 16 IQR (CV) : 3 (0.3) | 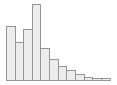 | 11739 (99.8%) |
| 11 | Sensation Seeking UPSS [numeric]  UPPS_Y_SS_SENSATION_SEEKING | Mean (sd) : 9.8 (2.7) min < med < max: 4 < 10 < 16 IQR (CV) : 4 (0.3) | 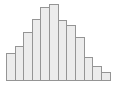 | 11739 (99.8%) |
| 12 | Positive Urgency UPSS [numeric]  UPPS_Y_SS_POSITIVE_URGENCY | Mean (sd) : 8 (3) min < med < max: 4 < 8 < 16 IQR (CV) : 4 (0.4) | 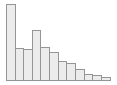 | 11739 (99.8%) |
| 13 | Lack of Perseverance UPSS [numeric]  UPPS_Y_SS_LACK_OF_PERSEVERANCE | Mean (sd) : 7 (2.3) min < med < max: 4 < 7 < 16 IQR (CV) : 3 (0.3) | 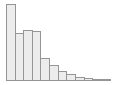 | 11739 (99.8%) |
| 14 | Behavioural Inhibition System [numeric]  Mean of  (BISBAS2_Y,  BISBAS3_Y,  BISBAS4_Y,  BISBAS6_Y) | Mean (sd) : 1.4 (0.7) min < med < max: 0 < 1.2 < 3 IQR (CV) : 1.2 (0.5) | 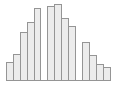 | 11740 (99.8%) |
| 15 | BAS Reward Responsiveness [numeric]  Mean of  (BISBAS8_Y,  BISBAS10_Y,  BISBAS11_Y,  BISBAS12_Y) | Mean (sd) : 2.2 (0.6) min < med < max: 0 < 2.2 < 3 IQR (CV) : 1 (0.3) | 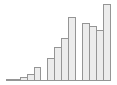 | 11739 (99.8%) |
| 16 | BAS Drive [numeric]  Mean of  (BISBAS13_Y,  BISBAS14_Y,  BISBAS15_Y,  BISBAS16_Y) | Mean (sd) : 1 (0.8) min < med < max: 0 < 1 < 3 IQR (CV) : 1 (0.7) | 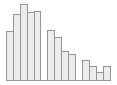 | 11739 (99.8%) |
| 17 | BAS Fun [numeric]  Means of  (BISBAS17_Y,  BISBAS18_Y,  BISBAS19_Y,  BISBAS20_Y) | Mean (sd) : 1.4 (0.7) min < med < max: 0 < 1.5 < 3 IQR (CV) : 0.8 (0.5) | 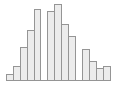 | 11739 (99.8%) |
