# Supplementary material for: Relationship between cognitive abilities and mental health as represented by cognitive abilities at the neural and genetic levels of analysis
Source: eLife. 2025 Nov 14;14:RP105537. doi: 10.7554/eLife.105537 (PMC12618009; doi:10.7554/eLife.105537)
Supplement: Supplementary file 3. — Med = Median IQR = interquartile range; CV = Coefficient of variation. Under the variable names, there are information about the method to compute these variables and the original variables names in ABCD data dictionary. [file elife-105537-supp3.docx]

Supplementary ﬁle 3. Summary statistics of the measures of socio-demographics, lifestyles and developmental adverse events in the baseline. Med = Median IQR = interquartile range; CV = Coefficient of variation. Under the variable names, there are information about the method to compute these variables and the original variables names in ABCD data dictionary.

| No | Variable | Stats / Values | Graph | Valid |
| --- | --- | --- | --- | --- |
| 1 | Bilingual Use [numeric]  if ACCULT_Q2_Y = 0 then 0  otherwise 11-(ACCULT_Q4_Y+ACCULT_Q5_Y) | Mean (sd) : 1 (1.7) min < med < max: 0 < 0 < 9 IQR (CV) : 1 (1.7) | 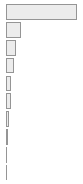 | 11681 (99.3%) |
| 2 | Parent Marital Status [factor]  DEMO_PRNT_MARITAL_V2 | 1. 1 2. 2 3. 3 4. 4 5. 5 6. 6 | 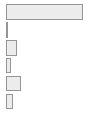 | 11667 (99.2%) |
| 3 | Parents’ Education [numeric]  Mean of  (DEMO_PRNT_ED_V2,  DEMO_PRTNR_ED_V2) | Mean (sd) : 16.4 (2.7) min < med < max: 3 < 17 < 21 IQR (CV) : 3.5 (0.2) | 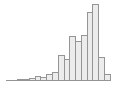 | 11748 (99.9%) |
| 4 | Parents’ Income [integer]  DEMO_COMB_INCOME_V2 | Mean (sd) : 7.2 (2.4) min < med < max: 1 < 8 < 10 IQR (CV) : 3 (0.3) | 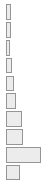 | 10756 (91.4%) |
| 5 | Household Size [integer]  DEMO_ROSTER_V2 | Mean (sd) : 4.7 (1.6) min < med < max: 0 < 4 < 19 IQR (CV) : 1 (0.3) | 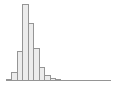 | 11484 (97.6%) |
| 6 | Economics Insecurities [numeric]  Sum of  (demo_fam_exp1_v2,  demo_fam_exp2_v2,  demo_fam_exp3_v2,  demo_fam_exp4_v2,  demo_fam_exp5_v2,  demo_fam_exp6_v2,  demo_fam_exp7_v2) | Mean (sd) : 0.5 (1.1) min < med < max: 0 < 0 < 7 IQR (CV) : 0 (2.3) | 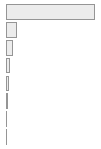 | 11628 (98.9%) |
| 7 | Area Deprivation Index [numeric]  RESHIST_ADDR1_ADI_WSUM | Mean (sd) : 94.7 (21) min < med < max: 1.1 < 98.8 < 125.7 IQR (CV) : 20.5 (0.2) | 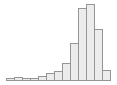 | 10889 (92.6%) |
| 8 | Lead Risk [numeric]  RESHIST_ADDR1_LEADRISK | Mean (sd) : 5.1 (3.1) min < med < max: 1 < 5 < 10 IQR (CV) : 6 (0.6) | 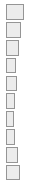 | 11112 (94.5%) |
| 9 | Uniform Crime Reports [numeric]  (RESHIST_ADDR1_GRNDTOT)/4 | Mean (sd) : 12.1 (5.8) min < med < max: 0 < 12.3 < 24.3 IQR (CV) : 5.8 (0.5) | 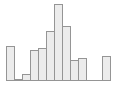 | 11114 (94.5%) |
| 10 | Parent reported Neighbourhood Safety [numeric]  Sum of  (NEIGHBORHOOD1R_P,  NEIGHBORHOOD2R_P,  NEIGHBORHOOD3R_P) | Mean (sd) : 11.7 (2.9) min < med < max: 3 < 12 < 15 IQR (CV) : 4 (0.3) | 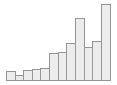 | 11715 (99.6%) |
| 11 | Child reported Neighbourhood Safety [numeric]  NEIGHBORHOOD_CRIME_Y | Mean (sd) : 4 (1.1) min < med < max: 1 < 4 < 5 IQR (CV) : 2 (0.3) | 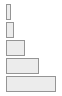 | 11738 (99.8%) |
| 12 | School Environment [numeric]  Sum of  (SCHOOL_2_Y,  SCHOOL_3_Y,  SCHOOL_4_Y,  SCHOOL_5_Y,  SCHOOL_6_Y,  SCHOOL_7_Y) | Mean (sd) : 19.9 (2.8) min < med < max: 6 < 20 < 24 IQR (CV) : 4 (0.1) | 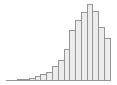 | 11735 (99.8%) |
| 13 | School Involvement [numeric]  Sum of  (SCHOOL_8_Y,  SCHOOL_9_Y,  SCHOOL_10_Y,  SCHOOL_12_Y) | Mean (sd) : 13.1 (2.4) min < med < max: 4 < 13 < 16 IQR (CV) : 3 (0.2) | 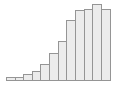 | 11736 (99.8%) |
| 14 | School Disengagement [numeric]  Sum of  (SCHOOL_15_Y,  SCHOOL_17_Y) | Mean (sd) : 3.7 (1.5) min < med < max: 2 < 4 < 8 IQR (CV) : 2 (0.4) | 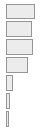 | 11737 (99.8%) |
| 15 | Parental Monitoring [numeric]  Mean of  (PARENT_MONITOR_Q1_Y, PARENT_MONITOR_Q2_Y,  PARENT_MONITOR_Q3_Y,  PARENT_MONITOR_Q4_Y,  PARENT_MONITOR_Q5_Y) | Mean (sd) : 4.4 (0.5) min < med < max: 1 < 4.4 < 5 IQR (CV) : 0.6 (0.1) | 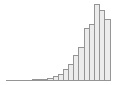 | 11739 (99.8%) |
| 16 | Parent reported Family Conflict [numeric]  Sum of  (FAM_ENVIRO1_P,  FAM_ENVIRO2R_P,  FAM_ENVIRO3_P,  FAM_ENVIRO4R_P,  FAM_ENVIRO5_P,  FAM_ENVIRO6_P,  FAM_ENVIRO7R_P,  FAM_ENVIRO8_P,  FAM_ENVIRO9R_P) | Mean (sd) : 2.5 (2) min < med < max: 0 < 2 < 9 IQR (CV) : 3 (0.8) | 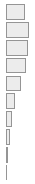 | 11750 (99.9%) |
| 17 | Child report Family Conflict [numeric]  Sum of  (FES_YOUTH_Q1,  FES_YOUTH_Q2,  FES_YOUTH_Q3,  FES_YOUTH_Q4,  FES_YOUTH_Q5,  FES_YOUTH_Q6,  FES_YOUTH_Q7,  FES_YOUTH_Q8,  FES_YOUTH_Q9) | Mean (sd) : 2 (2) min < med < max: 0 < 2 < 9 IQR (CV) : 3 (1) | 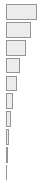 | 11735 (99.8%) |
| 18 | Parent reported Prosocial [numeric]  Mean of  (PROSOCIAL_Q1_P,  PROSOCIAL_Q2_P,  PROSOCIAL_Q3_P) | Mean (sd) : 1.7 (0.4) min < med < max: 0 < 2 < 2 IQR (CV) : 0.3 (0.2) | 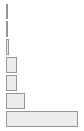 | 11700 (99.5%) |
| 19 | Child reported Prosocial [numeric]  Mean of  (PROSOCIAL_Q1_Y,  PROSOCIAL_Q2_Y,  PROSOCIAL_Q3_Y) | Mean (sd) : 1.7 (0.4) min < med < max: 0 < 1.7 < 2 IQR (CV) : 0.7 (0.2) | 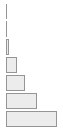 | 11729 (99.7%) |
| 20 | Tobbaco Before Pregnant [factor]  DEVHX_8_TOBACCO | 1. 0 2. 1 | 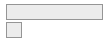 | 11487 (97.7%) |
| 21 | Tabbaco After Pregnant [factor]  DEVHX_9_TOBACCO | 1. 0 2. 1 | 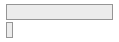 | 11501 (97.8%) |
| 22 | Alcohol Before Pregnant [factor]  DEVHX_8_ALCOHOL | 1. 0 2. 1 | 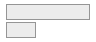 | 11084 (94.2%) |
| 23 | Alcohol After Pregnant [factor]  DEVHX_9_ALCOHOL | 1. 0 2. 1 | 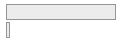 | 11473 (97.5%) |
| 24 | Marijuana Before Pregant [factor]  DEVHX_8_MARIJUANA | 1. 0 2. 1 | 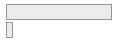 | 11428 (97.2%) |
| 25 | Marijuana After Pregant [factor]  DEVHX_9_MARIJUANA | 1. 0 2. 1 | 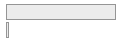 | 11489 (97.7%) |
| 26 | Lack of Sleep [numeric]  SLEEPDISTURB1_P | Mean (sd) : 1.7 (0.8) min < med < max: 1 < 2 < 5 IQR (CV) : 1 (0.5) | 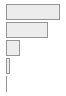 | 11757 (100.0%) |
| 27 | Sleep Disturbance [numeric]  SLEEPDISTURB2_P | Mean (sd) : 1.9 (1) min < med < max: 1 < 2 < 5 IQR (CV) : 1 (0.5) | 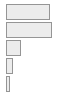 | 11757 (100.0%) |
| 28 | Sleep Initiating Maintaining [numeric]  SDS_P_SS_DIMS | Mean (sd) : 11.8 (3.7) min < med < max: 7 < 11 < 35 IQR (CV) : 4 (0.3) | 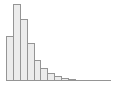 | 11757 (100.0%) |
| 29 | Sleep Breathing Disorders [numeric]  SDS_P_SS_SBD | Mean (sd) : 3.8 (1.2) min < med < max: 3 < 3 < 15 IQR (CV) : 1 (0.3) | 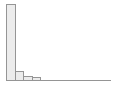 | 11757 (100.0%) |
| 30 | Sleep Arousal Disorders [numeric]  SDS_P_SS_DA | Mean (sd) : 3.4 (0.9) min < med < max: 3 < 3 < 15 IQR (CV) : 1 (0.3) | 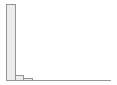 | 11757 (100.0%) |
| 31 | Sleep Wake Transition Disorders [numeric]  SDS_P_SS_SWTD | Mean (sd) : 8.2 (2.6) min < med < max: 6 < 7 < 30 IQR (CV) : 3 (0.3) | 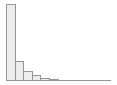 | 11730 (99.7%) |
| 32 | Sleep Excessive Somnolence [numeric]  SDS_P_SS_DOES | Mean (sd) : 6.9 (2.4) min < med < max: 5 < 6 < 25 IQR (CV) : 3 (0.4) | 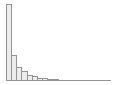 | 11756 (99.9%) |
| 33 | Sleep Hyperhidrosis [numeric]  SDS_P_SS_SHY | Mean (sd) : 2.4 (1.2) min < med < max: 2 < 2 < 10 IQR (CV) : 0 (0.5) | 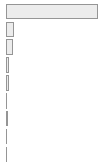 | 11757 (100.0%) |
| 34 | Individual Physical Extracurricular Activities [numeric]  Baseline sum of  (SAI_SS_SBOARD_PERWK_P, SAI_SS_CLIMB_PERWK_P,  SAI_SS_GYM_PERWK_P,  SAI_SS_ISKATE_PERWK_P,  SAI_SS_M_ARTS_PERWK_P,  SAI_SS_SKATE_PERWK_P,  SAI_SS_DANCE_PERWK_P,  SAI_SS_SURF_PERWK_P,  SAI_SS_TENNIS_PERWK_P,  SAI_SS_RUN_PERWK_P,  SAI_SS_MMA_PERWK_P,  SAI_SS_YOGA_PERWK_P) | Mean (sd) : 4.9 (5.6) min < med < max: 0 < 4 < 70 IQR (CV) : 8 (1.2) | 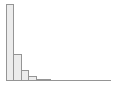 | 11671 (99.2%) |
| 35 | Team Physical Extracurricular Activities [numeric]  Baseline sum of  (SAI_SS_BASE_PERWK_P,  SAI_SS_BASKET_PERWK_P,  SAI_SS_FHOCK_PERWK_P,  SAI_SS_FBALL_PERWK_P,  SAI_SS_IHOCK_PERWK_P,  SAI_SS_POLO_PERWK_P,  SAI_SS_LAX_PERWK_P,  SAI_SS_RUGBY_PERWK_P,  SAI_SS_SOC_PERWK_P,  SAI_SS_WPOLO_PERWK_P,  SAI_SS_VBALL_PERWK_P) | Mean (sd) : 8.1 (7.6) min < med < max: 0 < 6 < 54 IQR (CV) : 13 (0.9) | 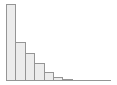 | 11653 (99.1%) |
| 36 | Non Physical Extracurricular Activities [numeric]  Baseline sum of  (SAI_SS_COLLECT_PERWK_P,  SAI_SS_MUSIC_PERWK_P,  SAI_SS_ART_PERWK_P,  SAI_SS_DRAMA_PERWK_P,  SAI_SS_CRAFTS_PERWK_P,  SAI_SS_CHESS_PERWK_P) | Mean (sd) : 4.9 (6.2) min < med < max: 0 < 4 < 54 IQR (CV) : 8 (1.3) | 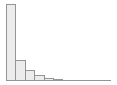 | 11646 (99.0%) |
| 37 | Physically Active [numeric]  PHYSICAL_ACTIVITY1_Y | Mean (sd) : 3.5 (2.3) min < med < max: 0 < 3 < 7 IQR (CV) : 3 (0.7) | 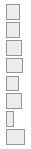 | 11734 (99.8%) |
| 38 | Mature Video Games Play [numeric]  SCREEN13_Y | Mean (sd) : 0.6 (0.9) min < med < max: 0 < 0 < 3 IQR (CV) : 1 (1.5) | 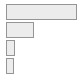 | 11742 (99.8%) |
| 39 | Mature Movies Watch [numeric]  SCREEN14_Y | Mean (sd) : 0.4 (0.6) min < med < max: 0 < 0 < 3 IQR (CV) : 1 (1.7) | 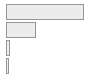 | 11741 (99.8%) |
| 40 | Weekday Screen Use [numeric]  Sum of  (SCREEN1_WKDY_Y,  SCREEN2_WKDY_Y,  SCREEN3_WKDY_Y,  SCREEN4_WKDY_Y,  SCREEN5_WKDY_Y,  SCREEN_WKDY_Y) | Mean (sd) : 3.5 (3.1) min < med < max: 0 < 2.5 < 24 IQR (CV) : 3.5 (0.9) | 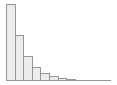 | 11725 (99.7%) |
| 41 | Weekend Screen Use [numeric]  Sum of  (SCREEN7_WKND_Y,  SCREEN8_WKND_Y,  SCREEN9_WKND_Y,  SCREEN10_WKND_Y,  SCREEN11_WKND_Y,  SCREEN12_WKND_Y) | Mean (sd) : 4.6 (3.6) min < med < max: 0 < 3.5 < 24 IQR (CV) : 4.2 (0.8) | 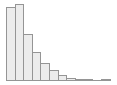 | 11720 (99.6%) |
| 42 | Developmental Prematurity [factor]  DEVHX_12A_P | 1. 0 2. 1 | 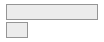 | 11618 (98.8%) |
| 43 | Birth Complications [numeric]  Sum of  (DEVHX_14A3_P,  DEVHX_14B3_P,  DEVHX_14C3_P,  DEVHX_14D3_P,  DEVHX_14E3_P,  DEVHX_14F3_P,  DEVHX_14G3_P,  DEVHX_14H3_P) | Mean (sd) : 0.4 (0.7) min < med < max: 0 < 0 < 8 IQR (CV) : 1 (2) | 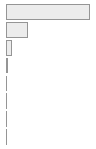 | 11007 (93.6%) |
| 44 | Pregnancy Complications [numeric]  Sum of  (DEVHX_10A3_P,  DEVHX_10B3_P,  DEVHX_10C3_P,  DEVHX_10D3_P,  DEVHX_10E3_P,  DEVHX_10F3_P,  DEVHX_10G3_P,  DEVHX_10H3_P,  DEVHX_10I3_P,  DEVHX_10J3_P,  DEVHX_10K3_P,  DEVHX_10L3_P) | Mean (sd) : 0.6 (1) min < med < max: 0 < 0 < 12 IQR (CV) : 1 (1.7) | 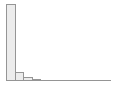 | 11027 (93.8%) |
