# Supplementary material for: Relationship between cognitive abilities and mental health as represented by cognitive abilities at the neural and genetic levels of analysis
Source: eLife. 2025 Nov 14;14:RP105537. doi: 10.7554/eLife.105537 (PMC12618009; doi:10.7554/eLife.105537)
Supplement: Supplementary file 4. — We only provided variables that were repeatedly correction in the follow-up here. Med = Median IQR = interquartile range; CV = Coefficient of variation. Under the variable names, there are information about the method to compute these variables and the original variables names in ABCD data dictionary. [file elife-105537-supp4.docx]

Supplementary ﬁle 4. Summary statistics of the measures of socio-demographics, lifestyles and developmental adverse events in the follow up. We only provided variables that were repeatedly correction in the follow up here. Med = Median IQR = interquartile range; CV = Coefficient of variation. Under the variable names, there are information about the method to compute these variables and the original variables names in ABCD data dictionary.

| No | Variable | Stats / Values | Graph | Valid |
| --- | --- | --- | --- | --- |
| 1 | Bilingual Use [numeric]  if ACCULT_Q2_Y = 0 then 0  otherwise 11-(ACCULT_Q4_Y+ACCULT_Q5_Y) | Mean (sd) : 1 (1.6) min < med < max: 0 < 0 < 9 IQR (CV) : 1 (1.6) | 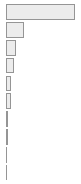 | 10768 (99.3%) |
| 2 | Parent Marital Status [factor]  DEMO_PRNT_MARITAL_V2_L | 1. 1 2. 2 3. 3 4. 4 5. 5 6. 6 | 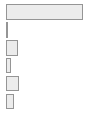 | 10766 (99.3%) |
| 3 | Parents’ Education [numeric]  Mean of  (DEMO_PRNT_ED_V2_2YR_L,  DEMO_PRTNR_ED_V2_2YR_L) | Mean (sd) : 17.7 (3.1) min < med < max: 1 < 18 < 23 IQR (CV) : 3 (0.2) | 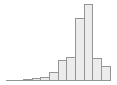 | 10823 (99.8%) |
| 4 | Parents’ Income [integer]  DEMO_COMB_INCOME_V2_L | Mean (sd) : 7.5 (2.3) min < med < max: 1 < 8 < 10 IQR (CV) : 2 (0.3) | 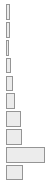 | 9953 (91.8%) |
| 5 | Economics Insecurities [numeric]  sum of  (demo_fam_exp1_v2_l,  demo_fam_exp2_v2_l,  demo_fam_exp3_v2_l,  demo_fam_exp4_v2_l,  demo_fam_exp5_v2_l,  demo_fam_exp6_v2_l,  demo_fam_exp7_v2_l) | Mean (sd) : 0.4 (1) min < med < max: 0 < 0 < 7 IQR (CV) : 0 (2.5) | 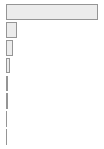 | 10734 (99.0%) |
| 6 | Parent reported Neighbourhood Safety [numeric]  Sum of  (NEIGHBORHOOD1R_P,  NEIGHBORHOOD2R_P,  NEIGHBORHOOD3R_P) | Mean (sd) : 11.6 (2.8) min < med < max: 3 < 12 < 15 IQR (CV) : 4 (0.2) | 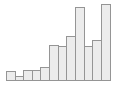 | 10752 (99.2%) |
| 7 | Child reported Neighbourhood Safety [numeric]  NEIGHBORHOOD_CRIME_Y | Mean (sd) : 4.1 (1) min < med < max: 1 < 4 < 5 IQR (CV) : 1 (0.2) | 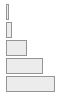 | 10811 (99.7%) |
| 8 | School Environment [numeric]  Sum of  (SCHOOL_2_Y,  SCHOOL_3_Y,  SCHOOL_4_Y,  SCHOOL_5_Y,  SCHOOL_6_Y,  SCHOOL_7_Y) | Mean (sd) : 19.6 (2.8) min < med < max: 6 < 20 < 24 IQR (CV) : 4 (0.1) | 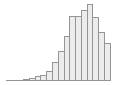 | 10808 (99.7%) |
| 9 | School Involvement [numeric]  Sum of  (SCHOOL_8_Y,  SCHOOL_9_Y,  SCHOOL_10_Y,  SCHOOL_12_Y) | Mean (sd) : 12.6 (2.3) min < med < max: 4 < 13 < 16 IQR (CV) : 3 (0.2) | 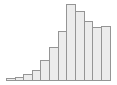 | 10808 (99.7%) |
| 10 | School Disengagement [numeric]  Sum of  (SCHOOL_15_Y,  SCHOOL_17_Y) | Mean (sd) : 4 (1.3) min < med < max: 2 < 4 < 8 IQR (CV) : 2 (0.3) | 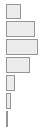 | 10808 (99.7%) |
| 11 | Parental Monitoring [numeric]  Mean of  (PARENT_MONITOR_Q1_Y, PARENT_MONITOR_Q2_Y,  PARENT_MONITOR_Q3_Y,  PARENT_MONITOR_Q4_Y,  PARENT_MONITOR_Q5_Y) | Mean (sd) : 4.5 (0.5) min < med < max: 1 < 4.6 < 5 IQR (CV) : 0.6 (0.1) | 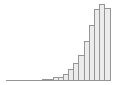 | 10811 (99.7%) |
| 12 | Parent reported Family Conflict [numeric]  Sum of  (FAM_ENVIRO1_P,  FAM_ENVIRO2R_P,  FAM_ENVIRO3_P,  FAM_ENVIRO4R_P,  FAM_ENVIRO5_P,  FAM_ENVIRO6_P,  FAM_ENVIRO7R_P,  FAM_ENVIRO8_P,  FAM_ENVIRO9R_P) | Mean (sd) : 2.4 (2) min < med < max: 0 < 2 < 9 IQR (CV) : 3 (0.8) | 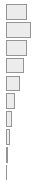 | 10813 (99.8%) |
| 13 | Child report Family Conflict [numeric]  Sum of  (FES_YOUTH_Q1,  FES_YOUTH_Q2,  FES_YOUTH_Q3,  FES_YOUTH_Q4,  FES_YOUTH_Q5,  FES_YOUTH_Q6,  FES_YOUTH_Q7,  FES_YOUTH_Q8,  FES_YOUTH_Q9) | Mean (sd) : 1.9 (1.8) min < med < max: 0 < 1 < 9 IQR (CV) : 3 (1) | 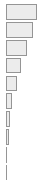 | 10268 (94.7%) |
| 14 | Parent reported Prosocial [numeric]  Mean of  (PROSOCIAL_Q1_P,  PROSOCIAL_Q2_P,  PROSOCIAL_Q3_P) | Mean (sd) : 1.7 (0.4) min < med < max: 0 < 2 < 2 IQR (CV) : 0.3 (0.2) | 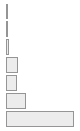 | 10788 (99.5%) |
| 15 | Child reported Prosocial [numeric]  Mean of  (PROSOCIAL_Q1_Y,  PROSOCIAL_Q2_Y,  PROSOCIAL_Q3_Y) | Mean (sd) : 1.7 (0.4) min < med < max: 0 < 2 < 2 IQR (CV) : 0.7 (0.2) | 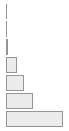 | 10811 (99.7%) |
| 16 | Lack of Sleep [numeric]  SLEEPDISTURB1_P | Mean (sd) : 2 (0.9) min < med < max: 1 < 2 < 5 IQR (CV) : 1 (0.4) | 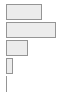 | 10803 (99.7%) |
| 17 | Sleep Disturbance [numeric]  SLEEPDISTURB2_P | Mean (sd) : 2.1 (1.1) min < med < max: 1 < 2 < 5 IQR (CV) : 2 (0.5) | 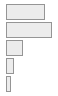 | 10803 (99.7%) |
| 18 | Sleep Initiating Maintaining [numeric]  SDS_P_SS_DIMS | Mean (sd) : 12 (3.8) min < med < max: 7 < 11 < 34 IQR (CV) : 5 (0.3) | 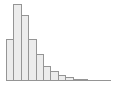 | 10803 (99.7%) |
| 19 | Sleep Breathing Disorders [numeric]  SDS_P_SS_SBD | Mean (sd) : 3.7 (1.2) min < med < max: 3 < 3 < 15 IQR (CV) : 1 (0.3) | 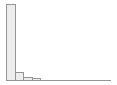 | 10803 (99.7%) |
| 20 | Sleep Arousal Disorders [numeric]  SDS_P_SS_DA | Mean (sd) : 3.3 (0.7) min < med < max: 3 < 3 < 11 IQR (CV) : 0 (0.2) | 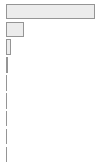 | 10803 (99.7%) |
| 21 | Sleep Wake Transition Disorders [numeric]  SDS_P_SS_SWTD | Mean (sd) : 7.8 (2.4) min < med < max: 6 < 7 < 28 IQR (CV) : 3 (0.3) | 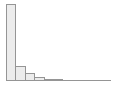 | 10802 (99.6%) |
| 22 | Sleep Excessive Somnolence [numeric]  SDS_P_SS_DOES | Mean (sd) : 7.1 (2.6) min < med < max: 5 < 6 < 25 IQR (CV) : 3 (0.4) | 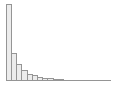 | 10803 (99.7%) |
| 23 | Sleep Hyperhidrosis [numeric]  SDS_P_SS_SHY | Mean (sd) : 2.3 (1) min < med < max: 2 < 2 < 10 IQR (CV) : 0 (0.4) | 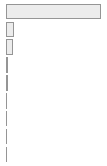 | 10803 (99.7%) |
| 24 | Individual Physical Extracurricular Activities [numeric]  Summation of  (SAI_SS_SBOARD_PERWK_P, SAI_SS_CLIMB_PERWK_P,  SAI_SS_GYM_PERWK_P,  SAI_SS_ISKATE_PERWK_P,  SAI_SS_M_ARTS_PERWK_P,  SAI_SS_SKATE_PERWK_P,  SAI_SS_DANCE_PERWK_P,  SAI_SS_SURF_PERWK_P,  SAI_SS_TENNIS_PERWK_P,  SAI_SS_RUN_PERWK_P,  SAI_SS_MMA_PERWK_P,  SAI_SS_YOGA_PERWK_P) in the baseline, and  (SAI_SS_SBOARD_PERWK_P_L,  SAI_SS_CLIMB_PERWK_P_L,  SAI_SS_GYM_PERWK_P_L,  SAI_SS_ISKATE_PERWK_P_L, SAI_SS_M_ARTS_PERWK_P_L,  SAI_SS_SKATE_PERWK_P_L,  SAI_SS_DANCE_PERWK_P_L, SAI_SS_SURF_PERWK_P_L, SAI_SS_TENNIS_PERWK_P_L, SAI_SS_RUN_PERWK_P_L, SAI_SS_MMA_PERWK_P_L, SAI_SS_YOGA_PERWK_P_L) in the one-year follow-up, and  (SAI_SS_SBOARD_PERWK_P_L, SAI_SS_CLIMB_PERWK_P_L, SAI_SS_GYM_PERWK_P_L, SAI_SS_ISKATE_PERWK_P_L, SAI_SS_M_ARTS_PERWK_P_L, SAI_SS_SKATE_PERWK_P_L , SAI_SS_DANCE_PERWK_P_L, SAI_SS_SURF_PERWK_P_L , SAI_SS_TENNIS_PERWK_P_L, SAI_SS_RUN_PERWK_P_L, SAI_SS_MMA_PERWK_P_L, SAI_SS_YOGA_PERWK_P_L) in the two-year follow-up. | Mean (sd) : 9.8 (10.5) min < med < max: 0 < 7 < 107 IQR (CV) : 16 (1.1) | 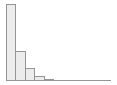 | 10416 (96.1%) |
| 25 | Team Physical Extracurricular Activities [numeric]  summation of  (SAI_SS_BASE_PERWK_P,  SAI_SS_BASKET_PERWK_P,  SAI_SS_FHOCK_PERWK_P,  SAI_SS_FBALL_PERWK_P,  SAI_SS_IHOCK_PERWK_P,  SAI_SS_POLO_PERWK_P,  SAI_SS_LAX_PERWK_P,  SAI_SS_RUGBY_PERWK_P,  SAI_SS_SOC_PERWK_P,  SAI_SS_WPOLO_PERWK_P,  SAI_SS_VBALL_PERWK_P) in the baseline, and  (SAI_SS_BASE_PERWK_P_L,  SAI_SS_BASKET_PERWK_P_L,  SAI_SS_FHOCK_PERWK_P_L,  SAI_SS_FBALL_PERWK_P_L,  SAI_SS_IHOCK_PERWK_P_L,  SAI_SS_POLO_PERWK_P_L,  SAI_SS_LAX_PERWK_P_L,  SAI_SS_RUGBY_PERWK_P_L,  SAI_SS_SOC_PERWK_P_L,  SAI_SS_WPOLO_PERWK_P_L, SAI_SS_VBALL_PERWK_P_L  ) in the one-year follow-up, and  ( SAI_SS_BASE_PERWK_P_L,  SAI_SS_BASKET_PERWK_P_L,  SAI_SS_FHOCK_PERWK_P_L,  SAI_SS_FBALL_PERWK_P_L,  SAI_SS_IHOCK_PERWK_P_L,  SAI_SS_POLO_PERWK_P_L,  SAI_SS_LAX_PERWK_P_L,  SAI_SS_RUGBY_PERWK_P_L,  SAI_SS_SOC_PERWK_P_L,  SAI_SS_WPOLO_PERWK_P_L, SAI_SS_VBALL_PERWK_P_L)  ) in the two-year follow-up | Mean (sd) : 18.2 (16.5) min < med < max: 0 < 15 < 90 IQR (CV) : 23 (0.9) | 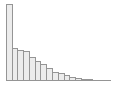 | 10405 (96.0%) |
| 26 | Non Physical Extracurricular Activities [numeric]  Summation of  (SAI_SS_COLLECT_PERWK_P,  SAI_SS_MUSIC_PERWK_P,  SAI_SS_ART_PERWK_P,  SAI_SS_DRAMA_PERWK_P,  SAI_SS_CRAFTS_PERWK_P,  SAI_SS_CHESS_PERWK_P) in the baseline and  (SAI_SS_COLLECT_PERWK_P_L,  SAI_SS_MUSIC_PERWK_P_L,  SAI_SS_ART_PERWK_P_L,  SAI_SS_DRAMA_PERWK_P_L,  SAI_SS_CRAFTS_PERWK_P_L,  SAI_SS_CHESS_PERWK_P_L) in the  One-year follow-up and  (SAI_SS_COLLECT_PERWK_P_L,  SAI_SS_MUSIC_PERWK_P_L,  SAI_SS_ART_PERWK_P_L,  SAI_SS_DRAMA_PERWK_P_L,  SAI_SS_CRAFTS_PERWK_P_L, SAI_SS_CHESS_PERWK_P_L) in the two-year follow-up) | Mean (sd) : 14 (14) min < med < max: 0 < 11 < 90 IQR (CV) : 21 (1) | 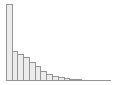 | 10390 (95.8%) |
| 27 | Physically Active [numeric]  PHYSICAL_ACTIVITY1_Y | Mean (sd) : 3.8 (2.2) min < med < max: 0 < 4 < 7 IQR (CV) : 3 (0.6) | 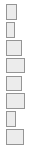 | 10813 (99.8%) |
| 28 | Mature Video Games Play [numeric]  SCREEN13_Y | Mean (sd) : 0.6 (0.9) min < med < max: 0 < 0 < 3 IQR (CV) : 1 (1.4) | 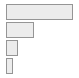 | 10812 (99.7%) |
| 29 | Mature Movies Watch [numeric]  SCREEN14_Y | Mean (sd) : 0.5 (0.7) min < med < max: 0 < 0 < 3 IQR (CV) : 1 (1.3) | 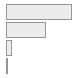 | 10804 (99.7%) |
